# 5,12-Dimethyl­pyrazino­[1,2-*a*:4,5-*a*′]dibenzimidazole-5,12-diium dichloride dihydrate

**DOI:** 10.1107/S1600536812046594

**Published:** 2012-11-24

**Authors:** Jie Han, Ming-gao Zhao, Jun Zhang, Lan Ma, Guang Fan

**Affiliations:** a Research Institute of Shaanxi Yanchang Petroleum (Group) Co. Ltd, Ke Ji No. 2 Road 75, Xi’an 710075, Shaanxi, People’s Republic of China; b Department of Pharmacology, School of Pharmacy, Forth Military Medical University, Chang-le West Road 17, Xi’an 710032, Shaanxi, People’s Republic of China; c College of Chemistry & Chemical Engineering, Xianyang Normal University, Xianyang 712000, Shaanxi, People’s Republic of China

## Abstract

The title hydrated salt, C_18_H_18_N_4_^2+^·2Cl^−^·2H_2_O, sits about an inversion centre, such that the asymmetric unit contains one half-mol­ecule. In the crystal, hydrogen bonds occur between the water mol­ecules and chloride anions, and there is π–π stacking of the benzene and imidazole rings of inversion-related pairs of mol­ecules, with a centroid–centroid distance of 3.704 (17) Å.

## Related literature

For descriptions of clinical applications of the benzimidazole ring system, see: Harrell *et al.* (2004 ▶). For a related structure, see: Sun *et al.* (2010 ▶).
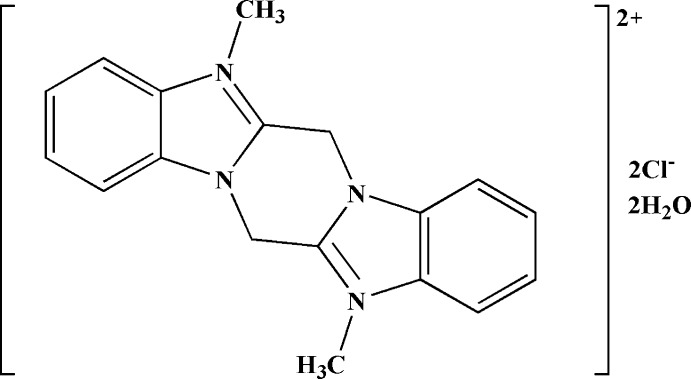


## Experimental

### 

#### Crystal data

C_18_H_18_N_4_^2+^·2Cl^−^·2H_2_O
*M* *_r_* = 397.30
Monoclinic, 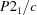

*a* = 8.1080 (12) Å
*b* = 9.0857 (14) Å
*c* = 12.9188 (19) Å
β = 94.426 (2)°
*V* = 948.8 (2) Å^3^
*Z* = 2
Mo *K*α radiation
μ = 0.36 mm^−1^
*T* = 296 K
0.38 × 0.28 × 0.17 mm

#### Data collection

Bruker SMART APEXII CCD diffractometer
Absorption correction: multi-scan (*SADABS*; Bruker, 2002 ▶) *T*_min_ = 0.876, *T*_max_ = 0.943
4604 measured reflections
1681 independent reflections
1371 reflections with *I* > 2σ(*I*)
*R*_int_ = 0.021

#### Refinement

*R*[*F* ^2^ > 2σ(*F* ^2^)] = 0.038
*wR*(*F* ^2^) = 0.114
*S* = 1.07
1681 reflections
127 parameters
2 restraints
H atoms treated by a mixture of independent and constrained refinement
Δρ_max_ = 0.16 e Å^−3^
Δρ_min_ = −0.20 e Å^−3^

### 

Data collection: *APEX2* (Bruker, 2002 ▶); cell refinement: *SAINT* (Bruker, 2002 ▶); data reduction: *SAINT*; program(s) used to solve structure: *SHELXS97* (Sheldrick, 2008 ▶); program(s) used to refine structure: *SHELXL97* (Sheldrick, 2008 ▶); molecular graphics: *SHELXTL* (Sheldrick, 2008 ▶); software used to prepare material for publication: *SHELXTL*.

## Supplementary Material

Crystal structure: contains datablock(s) I, global. DOI: 10.1107/S1600536812046594/pk2446sup1.cif

Structure factors: contains datablock(s) I. DOI: 10.1107/S1600536812046594/pk2446Isup2.hkl

Supplementary material file. DOI: 10.1107/S1600536812046594/pk2446Isup3.cml

Additional supplementary materials:  crystallographic information; 3D view; checkCIF report

## Figures and Tables

**Table 1:** Hydrogen-bond geometry (Å, °)

*D*—H⋯*A*	*D*—H	H⋯*A*	*D*⋯*A*	*D*—H⋯*A*
O1—H1*C*⋯Cl1^i^	0.83	2.33	3.1558 (19)	170
O1—H1*D*⋯Cl1^ii^	0.83	2.37	3.190 (2)	170

Symmetry codes: (i) 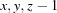; (ii) 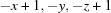.

**Fig. 1.**
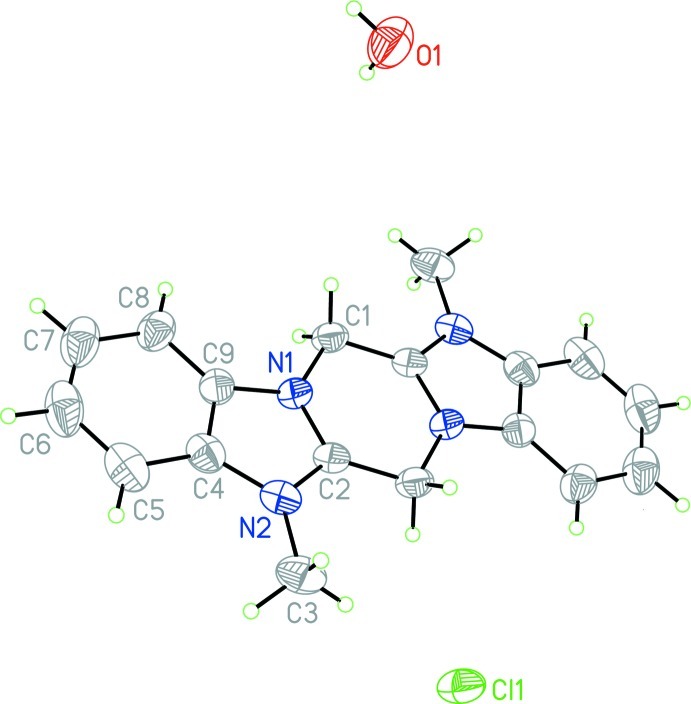
A view of the molecular structure of title compound. Displacement ellipsoids are drawn at the 35% probability level. Unlabelled atoms are related by inversion (1-x, -y, 1-z) to their labelled counterparts.

**Fig. 2.**
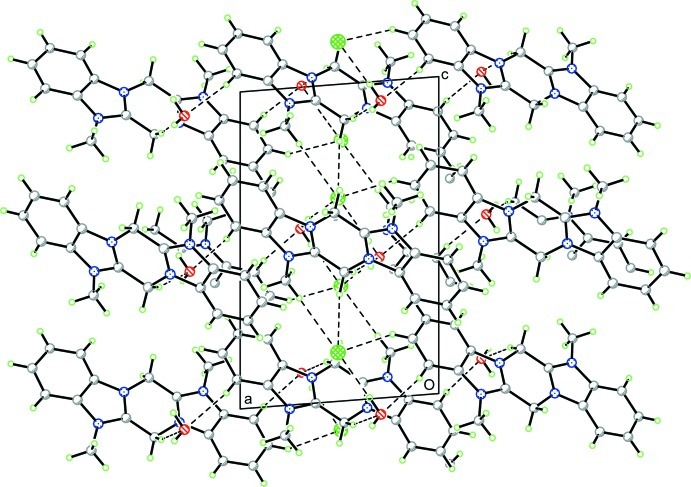
A three dimensional stacking diagram of (1) viewed down the b axis.

## supplementary crystallographic information

### Comment

Bis-benzimidazoles are DNA-minor grove binding agents that possess anti-tumor activity. The benzimidazole ring system is present in clinically approved antihistamines, antivirals, anthelmintics, and antiulcer medications (Harrell *et al.*, 2004). In addition to their biological activity, there are numerous other studies, including coordination and corrosion inhibitor abilities of benzimidazoles. Bis-benzimidazoles are also strong chelating agents (Sun *et al.*, 2010). Some of these derivatives are used as photographic materials and dyes. As part of our ongoing investigation of benzimidazole derivatives, the title compound was synthesized and its crystal structure is reported herein.

### Experimental

*N*-methylbenzene-1,2-diamine (2.5 mol), 2-chloroacetic acid (3 mmol), polyphosphoric acid (10 ml) and silica gel (1 g) were mixed and introduced in an open Erlenmeyer flask. The reaction mixture was irradiated in a domestic microwave oven for 3 min. After cooling to room temperature, methanol was added (20 ml) and the reaction mixture filtered. The filtrate was evaporated to dryness and subjected to column chromatography (10% hexane/ethyl acetate) to give green needle-like crystals of the title compound.

### Refinement

All H atoms attached to C atoms were generated in idealized positions and constrained to ride on their parent atoms, with C—H = 0.96 Å (methyl) and 0.93 Å (aromatic) with *U*_iso_(H) = 1.2*U*_eq_(C, aromatic) and *U*_iso_(H) = 1.5*U*_eq_(C, methyl). H atoms of water molecules were located in a difference Fourier map and refined with 1,2 and 1,3 distance restraints of 0.85 (2) Å and 1.39 (2) Å.

### Crystal data

**Table:**

C_18_H_18_N_4_^2+^·2Cl^−^·2H_2_O	*F*(000) = 416
*M**_r_* = 397.30	*D*_x_ = 1.391 Mg m^−^^3^
Monoclinic, *P*2_1_/*c*	Mo *K*α radiation, λ = 0.71073 Å
Hall symbol: -P 2ybc	Cell parameters from 1814 reflections
*a* = 8.1080 (12) Å	θ = 2.5–26.8°
*b* = 9.0857 (14) Å	µ = 0.36 mm^−^^1^
*c* = 12.9188 (19) Å	*T* = 296 K
β = 94.426 (2)°	Block, white
*V* = 948.8 (2) Å^3^	0.38 × 0.28 × 0.17 mm
*Z* = 2	

### Data collection

**Table:**

Bruker SMART APEXII CCD diffractometer	1681 independent reflections
Radiation source: fine-focus sealed tube	1371 reflections with *I* > 2σ(*I*)
Graphite monochromator	*R*_int_ = 0.021
φ and ω scans	θ_max_ = 25.1°, θ_min_ = 2.5°
Absorption correction: multi-scan (*SADABS*; Bruker, 2002)	*h* = −9→9
*T*_min_ = 0.876, *T*_max_ = 0.943	*k* = −10→10
4604 measured reflections	*l* = −10→15

### Refinement

**Table:**

Refinement on *F*^2^	Primary atom site location: structure-invariant direct methods
Least-squares matrix: full	Secondary atom site location: difference Fourier map
*R*[*F*^2^ > 2σ(*F*^2^)] = 0.038	Hydrogen site location: inferred from neighbouring sites
*wR*(*F*^2^) = 0.114	H atoms treated by a mixture of independent and constrained refinement
*S* = 1.07	*w* = 1/[σ^2^(*F*_o_^2^) + (0.0587*P*)^2^ + 0.1961*P*] where *P* = (*F*_o_^2^ + 2*F*_c_^2^)/3
1681 reflections	(Δ/σ)_max_ < 0.001
127 parameters	Δρ_max_ = 0.16 e Å^−^^3^
2 restraints	Δρ_min_ = −0.20 e Å^−^^3^

### Special details

**Table:**

Geometry. All e.s.d.'s (except the e.s.d. in the dihedral angle between two l.s. planes) are estimated using the full covariance matrix. The cell e.s.d.'s are taken into account individually in the estimation of e.s.d.'s in distances, angles and torsion angles; correlations between e.s.d.'s in cell parameters are only used when they are defined by crystal symmetry. An approximate (isotropic) treatment of cell e.s.d.'s is used for estimating e.s.d.'s involving l.s. planes.
Refinement. Refinement of *F*^2^ against ALL reflections. The weighted *R*-factor *wR* and goodness of fit *S* are based on *F*^2^, conventional *R*-factors *R* are based on *F*, with *F* set to zero for negative *F*^2^. The threshold expression of *F*^2^ > σ(*F*^2^) is used only for calculating *R*-factors(gt) *etc*. and is not relevant to the choice of reflections for refinement. *R*-factors based on *F*^2^ are statistically about twice as large as those based on *F*, and *R*- factors based on ALL data will be even larger.

### Fractional atomic coordinates and isotropic or equivalent isotropic displacement parameters (Å^2^)

**Table:**

	*x*	*y*	*z*	*U*_iso_*/*U*_eq_	
Cl1	0.49232 (8)	0.17546 (7)	0.85285 (4)	0.0755 (3)	
N1	0.35268 (18)	0.00311 (15)	0.44209 (10)	0.0454 (4)	
N2	0.2397 (2)	0.17478 (16)	0.53127 (12)	0.0521 (4)	
O1	0.7107 (3)	0.0906 (2)	0.05725 (17)	0.0961 (6)	
C1	0.4743 (2)	−0.0970 (2)	0.40528 (14)	0.0532 (5)	
H1A	0.5002	−0.0687	0.3360	0.064*	
H1B	0.4301	−0.1962	0.4021	0.064*	
C2	0.3745 (2)	0.09265 (19)	0.52381 (13)	0.0462 (4)	
C3	0.2198 (3)	0.2890 (2)	0.60909 (19)	0.0701 (6)	
H3A	0.2912	0.2682	0.6701	0.105*	
H3B	0.1069	0.2908	0.6268	0.105*	
H3C	0.2482	0.3830	0.5815	0.105*	
C4	0.1255 (2)	0.1382 (2)	0.44954 (16)	0.0557 (5)	
C5	−0.0348 (3)	0.1869 (3)	0.4213 (2)	0.0725 (7)	
H5	−0.0854	0.2590	0.4590	0.087*	
C6	−0.1142 (3)	0.1222 (3)	0.3345 (2)	0.0825 (8)	
H6	−0.2216	0.1515	0.3136	0.099*	
C7	−0.0399 (3)	0.0149 (3)	0.2772 (2)	0.0805 (7)	
H7	−0.0981	−0.0247	0.2188	0.097*	
C8	0.1178 (3)	−0.0338 (2)	0.30476 (16)	0.0642 (6)	
H8	0.1682	−0.1056	0.2667	0.077*	
C9	0.1975 (2)	0.0297 (2)	0.39233 (14)	0.0509 (5)	
H1C	0.653 (3)	0.101 (3)	0.0016 (12)	0.081 (8)*	
H1D	0.667 (4)	0.021 (2)	0.087 (2)	0.105 (11)*	

### Atomic displacement parameters (Å^2^)

**Table:**

	*U*^11^	*U*^22^	*U*^33^	*U*^12^	*U*^13^	*U*^23^
Cl1	0.0922 (5)	0.0875 (5)	0.0477 (3)	0.0113 (3)	0.0113 (3)	−0.0030 (2)
N1	0.0513 (9)	0.0469 (8)	0.0380 (8)	0.0018 (6)	0.0036 (6)	0.0004 (6)
N2	0.0584 (10)	0.0490 (9)	0.0508 (9)	0.0069 (7)	0.0152 (7)	0.0038 (6)
O1	0.0995 (15)	0.1007 (15)	0.0839 (14)	−0.0151 (12)	−0.0199 (11)	0.0121 (12)
C1	0.0599 (12)	0.0587 (11)	0.0414 (9)	0.0051 (9)	0.0068 (8)	−0.0074 (8)
C2	0.0525 (11)	0.0475 (10)	0.0396 (9)	0.0030 (8)	0.0103 (8)	0.0028 (7)
C3	0.0826 (15)	0.0569 (12)	0.0742 (14)	0.0093 (10)	0.0278 (12)	−0.0077 (10)
C4	0.0539 (11)	0.0548 (10)	0.0593 (12)	0.0024 (9)	0.0102 (9)	0.0165 (9)
C5	0.0604 (13)	0.0688 (14)	0.0901 (17)	0.0111 (10)	0.0171 (12)	0.0306 (12)
C6	0.0567 (14)	0.0892 (17)	0.0993 (19)	−0.0038 (12)	−0.0093 (13)	0.0444 (16)
C7	0.0726 (16)	0.0880 (17)	0.0773 (16)	−0.0122 (13)	−0.0181 (13)	0.0283 (14)
C8	0.0688 (13)	0.0683 (13)	0.0538 (12)	−0.0082 (10)	−0.0063 (10)	0.0120 (10)
C9	0.0504 (11)	0.0549 (10)	0.0472 (10)	−0.0026 (8)	0.0023 (8)	0.0117 (8)

### Geometric parameters (Å, º)

**Table:**

N1—C2	1.334 (2)	C3—H3B	0.9600
N1—C9	1.389 (2)	C3—H3C	0.9600
N1—C1	1.448 (2)	C4—C9	1.388 (3)
N2—C2	1.333 (2)	C4—C5	1.394 (3)
N2—C4	1.390 (3)	C5—C6	1.382 (4)
N2—C3	1.462 (3)	C5—H5	0.9300
O1—H1C	0.830 (10)	C6—C7	1.389 (4)
O1—H1D	0.827 (10)	C6—H6	0.9300
C1—C2^i^	1.473 (3)	C7—C8	1.374 (3)
C1—H1A	0.9700	C7—H7	0.9300
C1—H1B	0.9700	C8—C9	1.385 (3)
C2—C1^i^	1.473 (3)	C8—H8	0.9300
C3—H3A	0.9600		
			
C2—N1—C9	108.69 (15)	H3A—C3—H3C	109.5
C2—N1—C1	126.20 (15)	H3B—C3—H3C	109.5
C9—N1—C1	124.93 (15)	C9—C4—N2	106.96 (16)
C2—N2—C4	108.25 (15)	C9—C4—C5	120.5 (2)
C2—N2—C3	125.56 (18)	N2—C4—C5	132.5 (2)
C4—N2—C3	126.12 (17)	C6—C5—C4	116.4 (2)
H1C—O1—H1D	104 (3)	C6—C5—H5	121.8
N1—C1—C2^i^	109.51 (14)	C4—C5—H5	121.8
N1—C1—H1A	109.8	C5—C6—C7	122.5 (2)
C2^i^—C1—H1A	109.8	C5—C6—H6	118.7
N1—C1—H1B	109.8	C7—C6—H6	118.7
C2^i^—C1—H1B	109.8	C8—C7—C6	121.4 (2)
H1A—C1—H1B	108.2	C8—C7—H7	119.3
N2—C2—N1	109.82 (16)	C6—C7—H7	119.3
N2—C2—C1^i^	125.99 (16)	C7—C8—C9	116.3 (2)
N1—C2—C1^i^	124.19 (15)	C7—C8—H8	121.8
N2—C3—H3A	109.5	C9—C8—H8	121.8
N2—C3—H3B	109.5	C8—C9—C4	122.85 (19)
H3A—C3—H3B	109.5	C8—C9—N1	130.88 (19)
N2—C3—H3C	109.5	C4—C9—N1	106.27 (16)
			
C2—N1—C1—C2^i^	3.6 (3)	N2—C4—C5—C6	−178.57 (19)
C9—N1—C1—C2^i^	178.11 (15)	C4—C5—C6—C7	−0.4 (3)
C4—N2—C2—N1	−0.86 (19)	C5—C6—C7—C8	0.6 (4)
C3—N2—C2—N1	−177.88 (17)	C6—C7—C8—C9	0.0 (3)
C4—N2—C2—C1^i^	179.73 (17)	C7—C8—C9—C4	−0.8 (3)
C3—N2—C2—C1^i^	2.7 (3)	C7—C8—C9—N1	178.01 (18)
C9—N1—C2—N2	1.22 (19)	N2—C4—C9—C8	179.65 (17)
C1—N1—C2—N2	176.48 (16)	C5—C4—C9—C8	1.1 (3)
C9—N1—C2—C1^i^	−179.35 (16)	N2—C4—C9—N1	0.56 (19)
C1—N1—C2—C1^i^	−4.1 (3)	C5—C4—C9—N1	−177.98 (17)
C2—N2—C4—C9	0.16 (19)	C2—N1—C9—C8	179.92 (19)
C3—N2—C4—C9	177.16 (17)	C1—N1—C9—C8	4.6 (3)
C2—N2—C4—C5	178.5 (2)	C2—N1—C9—C4	−1.08 (19)
C3—N2—C4—C5	−4.5 (3)	C1—N1—C9—C4	−176.42 (16)
C9—C4—C5—C6	−0.5 (3)		

Symmetry code: (i) −*x*+1, −*y*, −*z*+1.

### Hydrogen-bond geometry (Å, º)

**Table:**

*D*—H···*A*	*D*—H	H···*A*	*D*···*A*	*D*—H···*A*
O1—H1*C*···Cl1^ii^	0.83	2.33	3.1558 (19)	170
O1—H1*D*···Cl1^i^	0.83	2.37	3.190 (2)	170

Symmetry codes: (i) −*x*+1, −*y*, −*z*+1; (ii) *x*, *y*, *z*−1.
